# NCR negative group 3 innate lymphoid cell (NCR^−^ILC3) participates in abnormal pathology of lung in cigarette smoking‐induced COPD mice

**DOI:** 10.1002/iid3.816

**Published:** 2023-03-27

**Authors:** Shuyuan Chu, Libing Ma, Xia Yang, Bo Xiao, Yaxi Liang, Shaojie Zheng, Linqiao Li

**Affiliations:** ^1^ Laboratory of Respiratory Disease The Affiliated Hospital of Guilin Medical University Guilin Guangxi China; ^2^ Department of Respiratory and Critical Care Medicine The Affiliated Hospital of Guilin Medical University Guilin Guangxi China; ^3^ Department of General Practice The First Affiliated Hospital of Guangxi Medical University Nanning Guangxi China

**Keywords:** cigarette smoking, COPD, ILC3, NCR negative

## Abstract

**Background:**

Natural cytotoxicity receptor negative innate lymphoid cell (NCR^−^ILC3) involves into mucosal homeostasis, inflammation regulation and tissue remodeling. The proportion of NCR^−^ILC3 is increased in the lung of smokers with chronic obstructive pulmonary disease (COPD). However, there's still few understandings on the role of NCR^−^ILC3 in COPD pathogenesis.

**Methods:**

COPD mice were induced by cigarette smoking. The pathology in lung was detected in histology. The frequency of NCR^−^ILC3 (CD3‐CD45+RORγt+NkP46‐) from murine lung was detected using flow cytometry. IL‐17+RORγt+ double positive cells in lung were assessed by double immunofluorescence staining. The protein expressions of epithelial‐to‐mesenchymal transition (EMT) markers, namely E‐cadherin and Vimentin, were assessed using immunohistochemistry staining and western blotting.

**Results:**

The frequency of NCR^−^ILC3 in lung was higher in COPD group than controls. The IL‐17+RORγt+ cells in lung from COPD mice were more than controls. E‐cadherin expression was decreased but Vimentin expression was increased in lung of COPD mice, when compared with controls. The frequency of NCR^−^ILC3 in lung tissues were positively correlated with mean linear intercept in lung, destructive index in lung and EMT, respectively.

**Conclusions:**

NCR^−^ILC3 could contribute to emphysema and EMT in lung of cigarette smoking‐induced COPD, which will provide further understanding on COPD pathogenesis of immune response.

## Graphical abstract

In this study, we found an increased number of NCR^−^ILC3 in lung tissues of cigarette smoking‐induced COPD mice, accompanied by an enhanced emphysema and bronchial EMT in COPD mice. The number of NCR^−^ILC3 in lung tissues were positively correlated with emphysema and bronchial EMT, respectively. These findings indicated that NCR^−^ILC3 may play a pivotal role in emphysema and airway EMT of COPD.

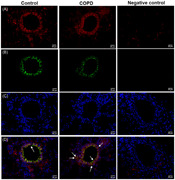

## 1 INTRODUCTION

Innate lymphoid cell (ILC) is a heterogeneous family of the innate immune system, which is composed of three groups, namely ILC1, ILC2, and ILC3, for their phenotypes and functions.^1^ Recently, ILC3s have attracted an increasing attention due to their roles in maintaining mucosal homeostasis, regulating inflammation, remodeling tissue and clearing pathogen. ILC3 is characterized by expressing retinoic acid receptor‐related orphan receptor γt (RORγt) for lineage specification. Most of ILC3 co‐expresses C‐C motif chemokine receptor 6 (CCR6), which is the receptor for epithelial chemokine CCL20.^2^, ^3^, ^4^, ^5^, ^6^ ILC3 can be further subdivided into natural cytotoxicity receptor positive (NCR^+^) ILC3 and NCR negative (NCR^−^) ILC3. The NCR is NKp44 in human, and is NKp46 in mice.^7^, ^8^ The NCR^−^ILC3 is a heterogeneous subgroup that also contains lymphoid tissue inducers (LTi) cells.^1^

Interestingly, for smokers with chronic obstructive pulmonary disease (COPD), proportions of ILC subsets in lung are skewed toward NCR^−^ILC3 whereas non‐COPD individuals have balanced proportions.^9^ The NCR^−^ILC3 also contained LTi cells.^9^ Moreover, NCR^−^ILC3s are enriched in lungs of severe COPD patients.^10^ Thus, NCR^−^ILC3 may participate in the COPD pathogenesis. However, there's still few understandings on the role of NCR^−^ILC3 in COPD pathogenesis.

The NCR^−^ILC3 could produce interleukin (IL)‐17 and IL‐22, whereas NCR^+^ILC3 produce IL‐22 but not IL‐17.^2^, ^3^, ^4^, ^5^, ^6^ IL‐17 plays a key role in the initiation of COPD. It mediates the recruitment of inflammatory cells in the lung and is essential for fibrosis of small airway in COPD.^11^ IL‐17 is also required for developing emphysema in response to cigarette smoking.^12^ Thus, NCR^−^ILC3 may contribute to COPD pathogenesis partly due to IL‐17. In this study, we explored the role of NCR^−^ILC3 in abnormal pathology of lung in COPD.

Moreover, ILC3 was reported to activate TGF‐β, which is a key mediator for tissue and mucosal repair and epithelial‐to‐mesenchymal transition (EMT) in airway.^13^ Our previous work found that IL‐17 could coordinate with cigarette to induce EMT of bronchial epithelial cells.^14^ Therefore, we also investigated the effect of IL‐17‐produced NCR^−^ILC3 on EMT of lung in cigarette smoking‐induced COPD mice in this study.

## 2 METHODS

### 2.1 Mice and cigarette smoke exposure

All mice used for experiments in this study were C57BL/6 background. Mice were maintained under pathogen‐free conditions. Eight to nine‐week‐old male mice were exposed to CS as previously described.^15^ Mice in COPD group was exposed to five cigarettes (Nanning Jiatianxia unfiltered cigarettes, 12 mg of tar and 0.9 mg of nicotine) four times every day with 30 min smoke‐free intervals in a closed 0.75 m^3^ room, 5 days per week for up to 20 weeks. The smoke:air ratio was 1:6. The controls were exposed to air. Each group included eight mice for administration and data analysis.

### 2.2 Ethical statement

All animal experiments were approved by the Institutional Animal Care and Use Committee of Guilin Medical University (approval number N/A), and according to the ARRIVE guideline.

### 2.3 Lung function measurements

Invasive pulmonary function of mice was tested with the forced oscillation technique using the FlexiVent system (Scireq) as described previously.^16^ Respiratory system elastance and compliance were captured using the Flexivent “Snapshot model.” Tissue elastance was captured with a constant phase model to obtain a parametric distinction between airway and tissue mechanics.

### 2.4 Histology, immunohistochemistry, and immunofluorescence staining

Mice were administrated terminal anesthesia with 2% isoflurane inhalation and then euthanized by cervical dislocation. Lungs were dissected out. One thirds of left lung inflated with fresh 4% paraformaldehyde was processed using a histological automatic tissue processor and embedded in paraffin. Three‐micrometer sections were cut and respectively administrated with hematoxylin and eosin (HE) stain, Periodic Acid‐Schiff (PAS) stain and Masson stain to evaluate airway inflammation, goblet cell hyperplasia and mucous secretion, and extracellular matrix in the lung tissues.^17^

As previously described,^18^, ^19^ the mean linear intercept (MLI) was obtained to evaluate airspace enlargement, and the destructive index was determined to assess alveolar destruction. Briefly, the MLI was obtained by dividing the length of a line drawn across the lung section by the total number of intercepts encountered in lung. The destructive index was revealed by the percentage of destroyed air spaces.

For immunohistochemistry, antigens in sections were retrieved via high temperature treatment and pressure cooker heating for 90 s in citrate buffer (pH 6.0). The sections were respectively incubated with rabbit antimouse α‐SMA (ab5694, Abcam Inc), rabbit antimouse IL‐17A (ab79056, Abcam Inc), rabbit anti‐mouse RORγt (orb385620; biorbyt), rat antimouse CCR6 (ab273580; Abcam Inc), rabbit antimouse CCL20 (orb676413; biorbyt), rabbit antimouse E‐cadherin (ab76319; Abcam Inc), and rabbit antimouse Vimentin (ab92547; Abcam Inc) primary antibodies. After that, they were incubated with polyclonal goat anti‐rabbit (ab6721; Abcam Inc), or goat anti‐rat (ab97057; Abcam Inc) IgG horseradish peroxidase (HRP)‐conjugated secondary antibody followed by incubation with diaminobenzidine liquid and peroxide buffer.

For immunofluorescence staining, the sections were incubated with rat anti‐mouse IL‐17A (ab118869; Abcam Inc), or rabbit anti‐mouse RORγt (orb385620; biorbyt) at 4°C overnight. Followed that, the slides were incubated with Rhodamine (TRITC)–conjugated Goat anti‐Rat IgG(H + L) (SA00007‐7; Proteintech) or Alexa fluor 488‐conjugated goat anti‐Rabbit IgG (SA00006‐2; Proteintech). The negative control was only incubated with the secondary antibodies. All micrographs were acquired on a microscope (BA210T; Motic).

### 2.5 Flow cytometry

The right lung was removed and washed in ice‐cold PBS. Single‐cell suspensions were prepared by gently disrupting the right lung. The cell suspension was washed twice with RPMI 1640, and mononuclear cells were then enriched from cell suspension. Cells were labeled by APC‐conjugated ani‐mouse CD3 (17‐0031‐82; eBioscience), PE‐conjugated anti‐mouse CD45 (12‐0451‐82; eBioscience), PE‐Cyanine7‐conjugated anti‐mouse NKp46 (25‐3351‐82; eBioscience) and Alexa Fluor™ 488‐conjugated anti‐mouse RORγt (53‐6981‐82; eBioscience). The gating method was negative control which wasn't labeled with fluorescence. All flow cytometry experiments were performed using CytoFLEX flow cytometer and the CytExpert software (Beckman Coulter).

### 2.6 Western blot analysis

Two thirds of left lung were subjected to western blot analysis as described.^18^ Briefly, lung tissues 40 mg were homogenized, and then centrifuged. The proteins were subjected to a 10% SDS‐polyacrylamide gel electrophoresis (SDS‐PAGE), followed by transferring onto PVDF membranes. The membranes were incubated with rabbit anti‐mouse E‐cadherin (ab76319; Abcam Inc), rabbit anti‐mouse Vimentin (ab92547; Abcam Inc), or rabbit anti‐mouse GAPDH (ab8245; Abcam Inc) primary antibodies at 4°C overnight, and then were incubated with HRP‐conjugated goat anti‐rabbit (ab205718, Abcam Inc) for 2 h at room temperature. The housekeeping protein was GAPDH. Finally, the blots were developed with the ECL Plus reagents (Thermo pierce).

### 2.7 Statistical analysis

Data were displayed as means ± Standard deviation (SD). Comparisons between groups were analyzed using unpaired *t*‐test. Correlation coefficients were calculated using Pearson's method. Results were considered statistically significant for *p* < .05. All statistical tests were performed using SPSS 21.0 (IBM SPSS Inc).

## 3 RESULTS

### 3.1 Pathological injury in lung tissues

**Figure 1:**
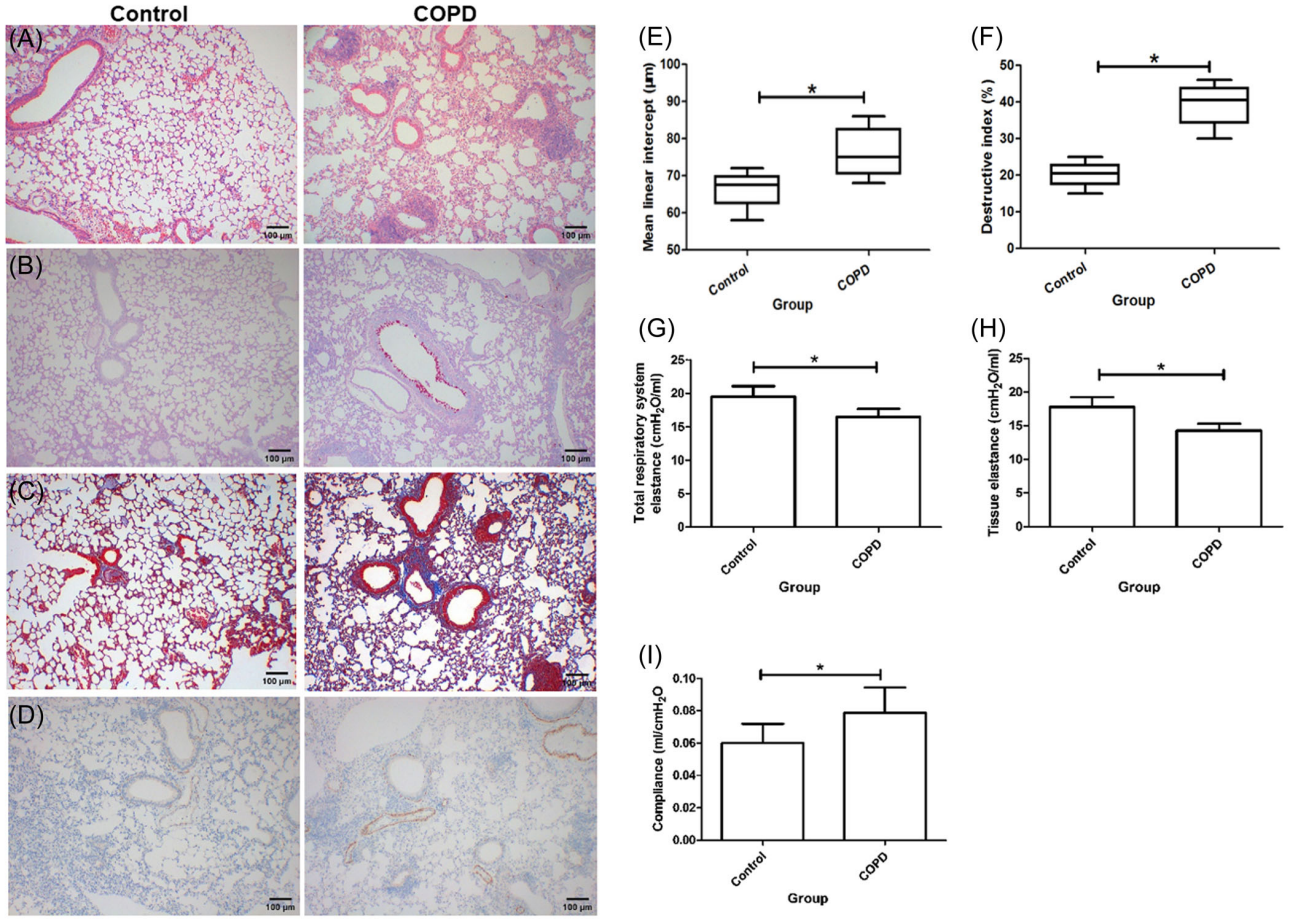
Histology and morphometric analysis of lung tissues and lung function (original magnification: ×100). (A) Hematoxylin and eosin stain. (B) Periodic Acid‐Schiff stain. (C) Masson stain. (D) α‐SMA+ cells. (E) Mean linear intercept. (F) Destructive index. (G) Total respiratory system elastance. (H) Tissue elastance. (I) Lung compliance. *p < .05.

In histology of lung tissues, COPD mice had increased inflammation (Figure 1A), goblet cell hyperplasia and mucous secretion (Figure 1B) extracellular matrix (Figure 1C), and smooth muscle thickening (Figure 1D), when compared with controls. That showed chronic and pathological injury in lung from COPD.

In morphology of lung, the MLI and destructive index in COPD mice were both increased when compared with controls (Figure 1E,F). Those showed more serious destruction of lung structure, particularly airspace with an enlargement, in COPD group than controls.

For lung function, COPD group had decreased total respiratory system elastance and tissue elastance, along with increased compliance, compared with control group (Figure 1G–I). That showed functional consequences in the model of COPD mice.

### 3.2 Frequency of NCR^−^ILC3 was increased in lung tissues from COPD mice

**Figure 2:**
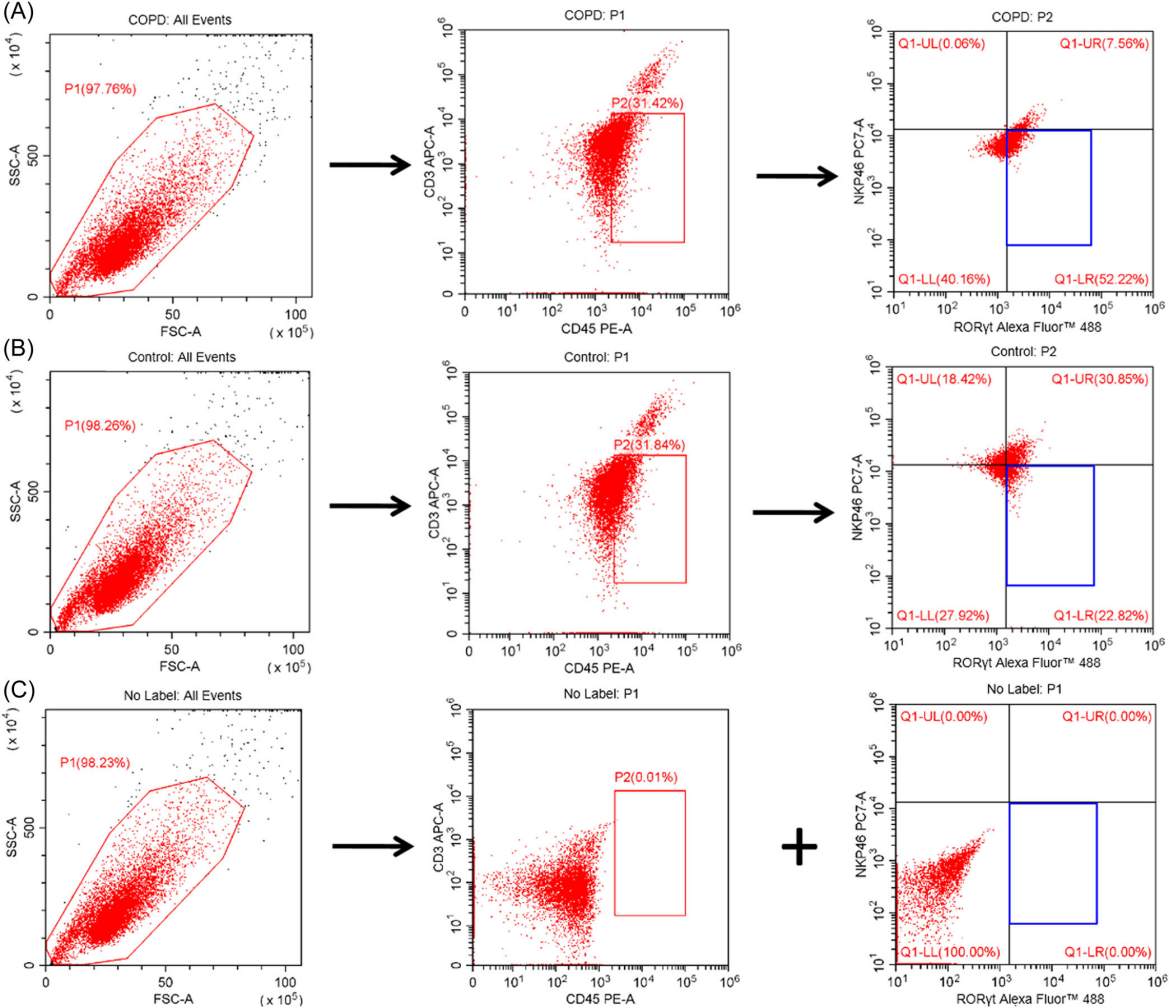
Flow cytometry of NCR^−^ILC3 in lung tissues. (A) chronic obstructive pulmonary disease group. (B) Control group. (C) No label for setting gates in flow cytometry.

The NCR^−^ILC3 was identified as CD3‐CD45+RORγt+NkP46‐ using flow cytometry. The frequency of NCR^−^ILC3 in lung tissues from COPD mice was higher than that from controls (COPD vs. controls = 52.59 ± 8.56% vs. 22.01 ± 6.45%) (Figure 2). This result showed an increase of NCR^−^ILC3 in lung from COPD mice.

### 3.3 Expressions of NCR^−^ILC3 related cytokines were increased in lung tissues from COPD mice

**Figure 3:**
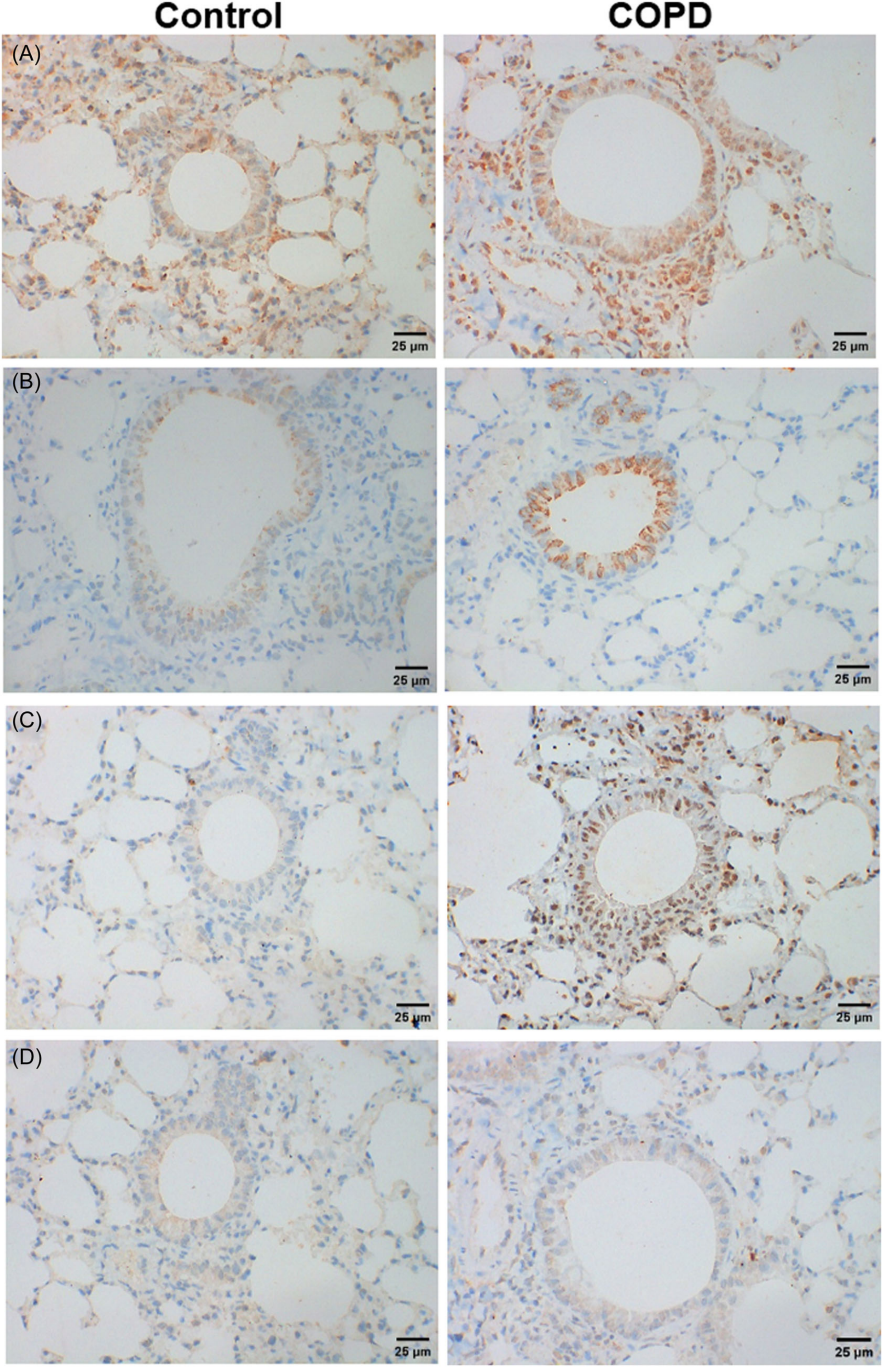
Immunohistochemistry staining of NCR^−^ILC3 related cytokines in lung tissues (original magnification: ×400). (A) IL‐17A+ cells. (B) RORγ+ cells. (C) CCR6+ cells. (D) CCL20+ cells.

The expressions of IL‐17, RORγt, CCR6, and CCL20 in lung tissues were explored by immunohistochemistry staining. All of IL‐17, RORγt, CCR6, and CCL20 were showed an increase of expression in lung tissues from COPD mice, when compared with controls (Figure 3).

**Figure 4:**
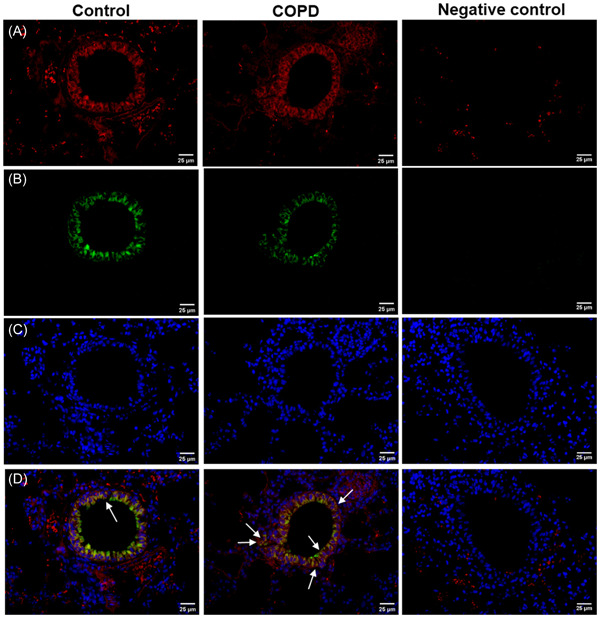
Immunofluorescence staining of IL‐17+RORγt+ double positive cells in lung tissues (original magnification: ×400). (A) IL‐17A+ cells. (B) RORγt+ cells. (C) DAPI. (D) Merge (A–C). The positive cells are indicated by arrows.

To explore the activity of NCR^−^ILC3 in small airway, the IL‐17+RORγt+ double positive cells in small airways was investigated using immunofluorescence staining. In COPD mice, IL‐17+RORγt+ cells were more than that in controls (Figure 4). The results support that NCR^−^ILC3 has more activity in lung of COPD mice than controls.

### 3.4 Epithelial‐mesenchymal transition (EMT) was increased in lung tissues from COPD mice

**Figure 5:**
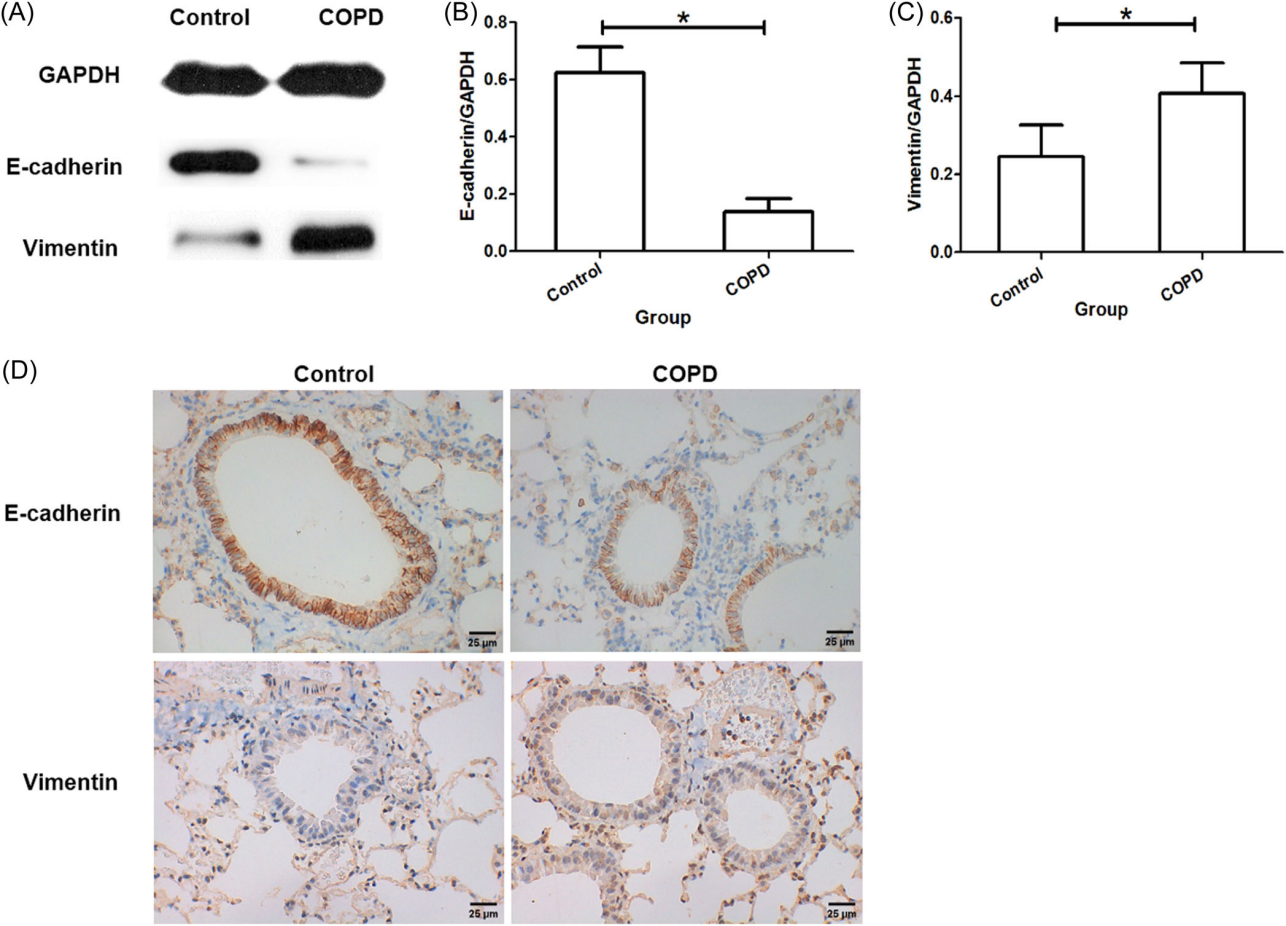
Expressions of E‐cadherin and Vimentin in lung tissues. (A) Western blotting. (B) Quantitation of protein bands for E‐cadherin. (C) Quantitation of protein bands for Vimentin. (D) Immunohistochemistry staining of E‐cadherin and Vimentin. *p < .05.

The protein expressions of E‐cadherin and Vimentin in lung tissues were assessed using western blotting. E‐cadherin protein expression was lower in COPD mice than that in controls, whereas Vimentin was expressed higher than that in controls (Figure 5A–C). Moreover, the immunohistochemistry staining of E‐cadherin and Vimentin was showed similar results as those from western blotting (Figure 5D). Those results suggested that COPD mice had an increased EMT in lung than controls.

### 3.5 Correlations

**Figure 6:**
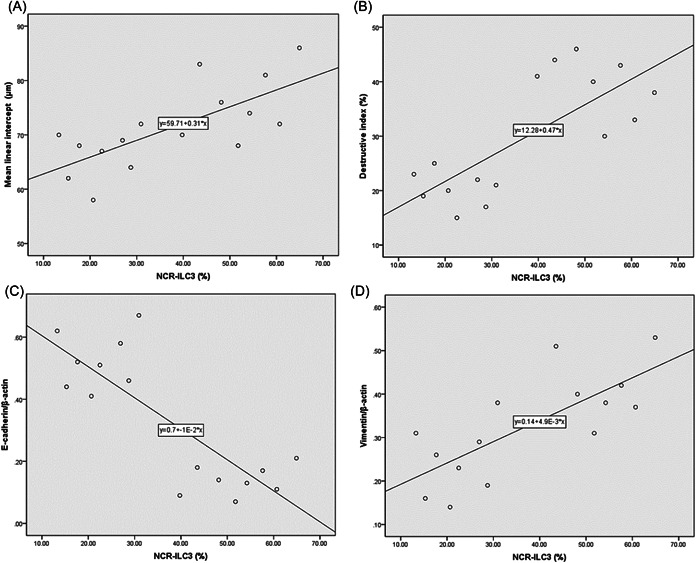
Correlations. Correlations between (A) NCR^−^ILC3 frequency and mean linear intercept, (B) NCR^−^ILC3 frequency and destructive index, (C) NCR^−^ILC3 frequency and protein expression of E‐cadherin, (D) NCR^−^ILC3 frequency and protein expression of Vimentin.

When all mice were considered together, the frequency of NCR^−^ILC3 in lung tissues was positively correlated with MLI (*r* = .718, *p* = .002), destructive index in lung (*r* = .758, *p* = .001) and protein expression of Vimentin in lung tissues (*r* = .751, *p* = .001), respectively (Figure 6A,B,D). In contrast, the frequency of NCR^−^ILC3 in lung tissues was negatively correlated with protein expression of E‐cadherin in lung tissues (*r* = −.818, *p* < .001) (Figure 6C).

## 4 DISCUSSION

Our present study confirmed an increase of NCR^−^ILC3 in lung tissues of cigarette smoking‐induced COPD mice, which were demonstrated to relate with emphysema and EMT in lung.

Our results showed that the frequency of NCR^−^ILC3 in lung tissues from COPD mice was higher than that from controls. And IL‐17+ cell, RORγt+ cell, CCR6+ cell and IL‐17+RORγt+ cell were more in lungs of COPD mice than controls, which may be related with an activation of NCR^−^ILC3 in response to cigarette smoking. Our findings were in consistent with previous studies on smokers with COPD patients,^9^, ^10^ and further demonstrated that cigarette smoking could induce an increase of NCR^−^ILC3 in lung of COPD. Thus, our results suggested that in COPD patients with cigarette smoking history, cigarette smoking may induce NCR^−^ILC3 in the occurrence and development of COPD.

In the present study, smoking‐induced COPD mice were showed significant emphysema in lungs. The MLI and destructive index confirmed the structure destruction in lung parenchyma of COPD mice. The lung function of COPD mice also demonstrated the pathological injury and functional decrease. The frequency of NCR^−^ILC3 in lung tissues was positively related with lung destruction in COPD mice, suggesting that NCR^−^ILC3 may participate in lung destruction in COPD. Since IL‐17 is crucial for emphysema development in response to cigarette smoking^12^ and NCR^−^ILC3 is one of the important sources of IL‐17,^20^ NCR^−^ILC3 may be involved into emphysema via producing IL‐17. Neutralization of IL‐17 may inhibit the effect of NCR^−^ILC3 on lung injury in COPD.

The EMT markers, namely E‐cadherin and Vimentin, were assessed in the lung of cigarette smoking‐induced COPD mice in our study. Our results showed that E‐cadherin protein expression was lower in COPD mice than that in controls, whereas Vimentin expression was higher than that in controls. The E‐cadherin+ cell and Vimentin+ cell distributions in small airways were in consistent with the protein expressions. Those results suggested that cigarette smoking could induce EMT in the lung of COPD mice, particularly in small airways, which was in consistent with previous findings.^21^

Furthermore, the EMT was positively correlated with the frequency of NCR^−^ILC3 in lung tissues of this present study, suggesting that NCR^−^ILC3 may be involved into airway EMT in COPD. That may be related with IL‐17, since IL‐17 could coordinate with cigarette to induce airway EMT.^14^ In addition, it may be associated with other mechanism, such as TGF‐β, which could be activated by ILC3.^13^ The precise mechanism of NCR^−^ILC3 in airway EMT of COPD should be further investigated in in vitro study in future.

We identified the NCR^−^ILC3 using markers as CD3‐CD45+RORγt+NkP46‐. RORγt has been found to express in immune cells including CD4 and CD8 double positive thymocytes in thymus,^22^ Th17,^23^ Tc17,^24^ regulatory T cells,^25^, ^26^ invariant natural killer T cells (iNKT),^27^ γδ T cells,^28^ NK cells^29^ and ILC3s.^1^ The negative CD3 markers could exclude T cells and iNKT. The negative NCR could exclude NK cells, since NCR is reliable marker for NK cells.^30^ Therefore, the markers CD3‐CD45+RORγt+NkP46‐ could identify NCR^−^ILC3 in our study. However, it should be aware that the NCR^−^ILC3 remains a heterogeneous group that also encompasses LTi cells.

## 5 CONCLUSION

In conclusion, NCR^−^ILC3 could contribute to emphysema and EMT in lung of cigarette smoking‐induced COPD, which will provide further understanding on COPD pathogenesis of immune response.

## AUTHOR CONTRIBUTIONS

**Shuyuan Chu**: Conceptualization (lead); methodology (lead); formal analysis (lead); writing—original draft (lead); writing—review and editing (equal)；funding acquisition (lead); investigation (lead). **Libing Ma**: Conceptualization (supporting); methodology (equal); writing—review and editing (equal). **Xia Yang**: Methodology (lead); formal analysis (equal); writing—review and editing (equal); funding acquisition (equal). **Bo Xiao**: Methodology (equal); writing—review and editing (equal). **Yaxi Liang**: Methodology (equal); writing—review and editing (equal). **Shaojie Zheng**: Methodology (equal); writing—review and editing (equal). **Linqiao Li**: Methodology (equal); writing—review and editing (equal).

## CONFLICT OF INTEREST STATEMENT

The authors declare no conflict of interest.

## Data Availability

The data that support the findings of this study are available from the corresponding author upon reasonable request.
